# An engineering review of external fixators

**DOI:** 10.1016/j.medengphy.2021.11.002

**Published:** 2021-12

**Authors:** P.L.N. Fernando, Aravinda Abeygunawardane, PCI Wijesinghe, Parakrama Dharmaratne, Pujitha Silva

**Affiliations:** a Centre for Biomedical Innovation, University of Moratuwa, Sri Lanka; b Department of Mechanical Engineering, University of Moratuwa, Sri Lanka; c Department of Materials Science and Engineering, University of Moratuwa, Sri Lanka; d National Hospital of Sri Lanka, Sri Lanka; e Consultant Orthopaedic Surgeon, Ministry of Health, Sri Lanka; f Department of Electronic and Telecommunications Engineering, University of Moratuwa, Sri Lanka

**Keywords:** Biomechanical testing, Composite materials, External fixators, Finite element modelling

## Abstract

External Fixators are a common technique used to treat a variety of issues related to bones, predominantly due to its non-intrusive nature and versatility in terms of form and materials. While it is mainly used to treat open fractures, its other uses include limb lengthening, deformity correction, bone grafting, compression of non-unions and stabilization of dislocations. Its earliest use dates as far back as 400 BCE and has undergone significant improvements, focusing on both customization and optimization. These two aspects highlight the significance of complementing the orthopaedic requirements with engineering knowledge and its applications. Hence, this review paper aims to conduct an examination of recent developments of external fixators with a special focus on its structure, the usage of materials and biomechanical investigations using experimental and numerical techniques. The paper presents the existing level of engineering knowledge with regards to these aspects and identifies research gaps, which can improve the quality of the commonly used external fixators.

## Highlights

• Mechanical stability plays a key role in the effectiveness of external fixators.
• Strength and stiffness are the main factors which contributes towards stability.
• Modified configurations of linear, circular and hybrid fixators are investigated.
• Light weight composite materials are gradually replacing traditional metallic alloys.
• Existing research gaps in further optimizing external fixators are identified.

## 1 Introduction

The use of External Fixators (EFs) in treating injuries dates all the way back to 400 BCE. Over many centuries, EFs have undergone significant changes, most of which have been due to either orthopaedic necessities or advancements in technical and manufacturing capabilities [1, 2].

EF is a common treatment technique used in the case of high-energy trauma related open bone fractures. From an orthopaedic point of view, EFs are used to facilitate wound care while allowing additional plastic or vascular surgery, as well as good visualisation of the fracture site when using radiography. All this must be achieved without compromising bone loss at the fracture site [3]. Depending on the expected nature and rate of healing, the period of using an EF could span from weeks to months. While the correct usage of EFs can lead to the healing of the fracture through the expected biological processes of bone regeneration, its improper use can severely hamper the quality of healing [4]. Hence, this highlights the importance of a sound understanding of the behaviour and performance of EFs.

An orthopaedic definition of bone fracture includes discontinuity of the bone, along with soft tissue injury and its healing is significantly influenced by the magnitude and distribution of mechanical stresses within the fracture bridging tissues [5]. EFs, which are a form of less invasive surgery, have been used as an alternative to internal fixators, which generally takes the form of plates with screws [6, 7]. Earliest studies, both in terms of surgical [8] and mechanical [9], identified the potential of EFs compared to plate fixation, especially in terms of its rate of healing and possible serious infections. EFs have been successfully used in fractures involving the humerus, forearm, pelvis, femur, tibia, and fibula [10], [11], [12], [13], [14]. It is also used for deformity correction, limb lengthening and treatment of bone defects [15, 16]. Therefore, from a purely orthopaedic point of view, previous studies support the use of EFs in treating bone fractures.

In its most basic form, EFs consist of a rod (or bar), on to which pins are clamped. The pins are in contact with the fractured bones which require healing, where as other parts such as rods and clamps do not directly come into contact with the tissues. All these components contribute towards the mechanical stability of the bone-EF system [17].

The main expectations of an EF frame include being safe, non-obstructive, adaptable to a wide variety of injuries and stiff enough to maintain alignment. Moreover, it should allow full weight-bearing where needed and have a low rate of serious complications. According to Behrens and Searls [18], these goals can be achieved by adherence to three basic principles. In order of decreasing importance, these principles are; that the frame should not damage vital anatomical structures, it should provide sufficient access for debridement and secondary procedures, and lastly it should fulfil the mechanical demands of the patient and the injury. To this end, the mechanical stability of an EF plays a key role, where geometric and material characteristics must be taken into consideration.

From an engineering point of view, two key parameters which influence the mechanical stability of EFs is the stiffness and strength. EFs with high stiffness can have a negative effect towards healing of the fracture due to high stresses in the bones as well as in the pins [19]. However, too much flexibility in the mechanical stiffness of EFs can also lead to excessive movement of the EFs, as well as the bone, which can also lead to implant failure and poor healing of the fracture. In addition, from a clinical point of view, excessively flexible EFs can be hard to manage. Hence, it is apparent that a compromise needs to be made in terms of the flexibility of the EFs and this can be achieved either through executing structural changes to the EF unit or by a careful selection of materials. As for the strength, the strength of each individual component will contribute towards the overall strength of the EF. Maintaining lower stress levels in each component will ensure the components deform only elastically (as opposed to plastically), which in turn facilitates the reusability of EFs. Here again, the distribution of stresses within the EF, which is determined by the structure of the EF and the choice of materials play a significant role. Therefore, this paper will focus on three aspects, namely different structural systems of EFs (Section 2), the materials used in EFs (Section 3) and the biomechanical investigations carried out on the EFs (Section 4). To the best of the authors’ knowledge, there has not been a review paper which has looked into the latter two aspects, while the publications that have reviewed different EF structural configurations have all been before 2010 [20, 21]. Finally, the paper concludes by highlighting the existing research gaps (Section 5), by critiquing the present literature from both engineering and orthopaedic viewpoints, which can be pursued as potential future research directions to further optimize and customize the use of EFs.

The well-known research databases such as ScienceDirect and PubMed, and academic search engines such as Google Scholar and ResearchGate were explored to extract meaningful journal publications, using the keywords *external fixators, linear external fixators, circular external fixators, external fixator materials, biomechanics of external fixators, finite element modelling of external fixators* and similar. In terms of the year of publication, the scope of this initial search was set to after 2015 and the latest database search was conducted in December 2020. Only research papers published in English were considered. Further references were identified by referring to citations within the explored papers, some of which are considered fundamental papers in the field of external fixators. This review is not intended to provide a comprehensive list of publications which have studied EFs, but to study the state-of-the-art knowledge of EF design. Hence, the eligibility criteria to include these research papers in this review mainly depended on whether they provided new insights beyond traditional structural configurations, materials and biomechanical evaluations, related to external fixators. Research publications which were predominantly clinical studies in nature and studies which focused on the use of external fixators on animals were excluded from this review. The chosen papers were organized into three broad groups, to cover the three main aspects covered in this review. It must be noted that some papers provided information for more than one aspect and hence, were considered in the discussion of multiple sections. Within these three broad areas, the papers were discussed in such a way that a coherent argument could be developed. The direction taken in each of these Sections have been mentioned at the beginning of each Section.

## 2 Commonly used EF structural systems

This Section aims to identify the mechanical performance of different types of EFs based on their structure. As summarized in Table 1, over the centuries, the basic structure of EFs have undergone significant changes. From an orthopaedic point of view, these have been categorized into 6 generations.

**Table 1:** Classification of different structural configurations of EFs [22, 23, 24].

Generation	Name of EF	Main features
1st Generation	Unilateral frame	The basic form which was used as the basis for the subsequent EFs
2nd Generation	Uniplanar frame	Subjected to cantilever bending and functions as a bi-planar system
3rd Generation	Ring fixator (Ilizarov)	Superior biomechanical properties
4th Generation	Articulated fixator	The unilateral frame is modified to allow for a range of motions in the joint
5th Generation	Hexapod (Taylor Spatial Frame)	Functions as a multi-planar system with 6 degrees of freedom and can be used for deformity corrections too
6th Generation	Hybrid ring	Combines the advantage of metaphyseal fixation with ease of use of half pins but is not biomechanically superior to full ring

**Fig. 1:**
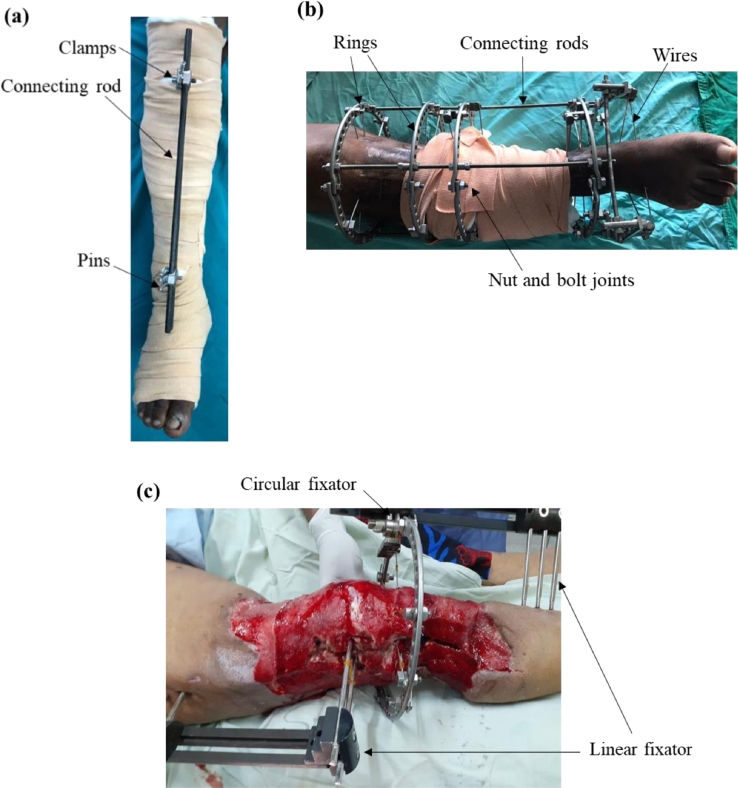
Illustrations of (a) linear, (b) circular and (c) hybrid fixators.

From a structural point of view, these EFs can be broadly categorized as either linear, circular or hybrid fixators, as shown in Fig. 1. The unilateral and uniplanar EFs fall under the former, while the ring and hexapod EFs fall under latter, with the hybrid ring configuration possessing qualities of both linear and circular types. There have been studies which have identified possible failures in EF systems such as instability of EFs, pin loosening, pin tract infection etc. [25, 26, 27], which highlights the need towards a clear understanding of the structural performance of EFs.

As evident by Table 1, orthopaedic surgeons have a range of EFs to choose from in treating different types of fractures. Generally, a unilateral EF is preferred in treating simple fractures such as spiral and oblique fractures. On the other hand, multiplanar EF configurations have found to be effective in treating more complex forms of fractures such as comminuted and pilon fractures, as well as in deformity correction surgeries [3]. The decision as to which structural form of EF is best suited for a given scenario is very much a biomechanical decision. To this end, two major factors to consider are the stability and the stiffness of the EF system.

Firstly, the stability of the EF will ensure a uniform distribution of stresses and displacement in the EF as well as the fracture site, which is significant in achieving bone healing without undesirable complications due to possible stress concentration and excessive movements. High stresses could severely hamper the bone formation at the fracture site, which can lead to either delayed bone union or in extreme cases bone non-union. The absence of fracture stability, which is a key mechanical prerequisite towards fracture healing, can lead to non-union at fracture site. Direct fracture healing occurs when the bone-to-bone contact of the fracture ends occur in correct alignment and rotation with absolute stability, while in the case of indirect fracture healing relative stability would suffice [28]. In the case of using EFs in trauma, non-union is a common complication due to its inadequate stability and can affect both the direct and indirect healing of the fracture. This is because in such cases, the micromotion between the fracture ends exceed the maximum allowable values of 2 µm and 10 µm, respectively [29, 30]. If left without regular review, it can lead to both atrophic and hypertrophic non-union, as well as pseudoarthrosis. Sancaktar et al. [31], carried out an experimental study to develop a reliable method of quantifying the force passing through the osteotomy site which is of significant interest, especially when external fixators are used in elective orthopaedic cases such as arthrodesis [32]. This is an important factor to prevent bone malalignment and avoid other problems related to the bone remodelling procedure, due to the effects of the EF. Generally, the osteotomy is the final step of the surgery after the fixation of the proximal and distal frames and hence, the surgeon may assume that the forces across the proximal and distal constructs cancel each other and that there will be no residual force across the site of osteotomy. However, in reality, once the osteotomy is completed, a residual force may pass through the site of osteotomy, which may lead to malalignment. Therefore, it is imperative to identify this issue and improve the frames, so as to minimize this form of complication. Moreover, the magnitude of stresses at the pin-bone interface could lead to its mechanical deterioration, which in turn could lead to complications such as pin loosening and pin tract infection [17]. This can affect the stability of the EF system. Therefore, the contact between the pin and the bone matters and hence, the angle of the pins can affect the stresses generated in the EF. In an ideal scenario, the pins are inserted perpendicularly into the bone while the connecting rod is placed parallel to the bone [33]. However, depending on the nature of the fracture, this arrangement may not always be possible from a clinical point of view. Another factor which was found to contribute towards the stability of the EF system is the distance between the connecting rod and the fractured bone, where a shorter distance improves the stability [34]. Moreover, several other factors such as increasing the number of pins on each fracture fragment, increasing the number of connecting rods and using multi-planar fixation were identified to increase the stability of EFs [20]. In fact, conducting an optimization study based on the displacement at the fracture focus, Roseiro et al. [5] recommended that while the first pin must be placed as close as possible to the fracture site, the location of the second pin depends on whether the dominant loading action is axial, bending or torsional. However, this work also highlighted that this arrangement of pins contradicts the widely accepted “near and far rule” by the orthopaedic surgeons, thus this finding requires further scrutiny. As for the displacement at the fracture site, it can be categorized as either primary or secondary [35]. Primary displacement is the loss of reduction of the fracture or osteotomy that occurs at the time of primary surgery and is mainly due to factors that reduce the stability of the construct. In elective cases such as osteotomy for bone lengthening or deformity correction, the imbalanced forces in the proximal and distal segments tend to pass through the site of least resistance, which is in fact the site of the osteotomy. Thus, immediately after the osteotomy, a certain degree of displacement may be observed at the site of the osteotomy. This can be especially observed in cases where the linear EF type, Limb Reconstruction System (LRS) or circular EFs are used, because the magnitude of the tensile force that passes through each pin could be significant in them. In the case of circular EFs, if there is an imbalance of forces in the proximal and distal rings, the bone ends will displace and attempt to balance it as soon as the osteotomy is complete. The secondary displacement at the fracture site is generally due to factors which affect the stability of the construct, when used for a prolonged period of time. The site of maximum stress points, which is at the pin–bone interface, should be assessed regularly to ensure its resistance to fatigue and deformity is adequate to withstand the multiple loading cycles across the construct during its intended period of use.

Secondly, for an EF to function effectively, its stiffness must match the forces and moments at the fracture site [18]. An overly rigid or overly flexible EF can induce undesirable levels of movement at the fracture site as well as stress shielding, which highlights the importance of identifying the optimum level of stability expected of an EF. Sternick et al. [36] identified that of all the components in an EF, the pins play a crucial role towards the stability of a basic EF. Several factors such as the angle of the pin, improper pin placement, pin diameter and the distance between the pin and the fracture gap can contribute towards the micromotions in the pins [3, 37]. Li et al. [38] identified that the influence of the deviation in the proximal and distal pins is correlated with the joint deviation, thereby providing a guideline for the physician in deciding on the adjustments required to the pins, during the period where the EF is in use. A key finding of this study identifies that the impact due to the movement of one pin, is not limited to its immediate fixator joint. This is particularly useful in scenarios where the proximal and distal pins vary with a difference in its magnitude. Giotakis and Narayan [39] recommended maintaining the size of the pin within the third of the diameter of the bone, to reduce the micromotions of pins. Basat et al. [40], identified that the stiffness of an EF system must be such that controlled movement is allowed especially along the primary axis, which is the one parallel to the fractured bone. Donaldson et al. [37] conducted a numerical study to investigate the influence of bone yielding towards pin loosening in an unilateral EF. Both a two-pin and three-pin arrangement was investigated under an axial compression load of 700 N on the fractured bone. It was found that the three-pin arrangement as opposed to a two-pin arrangement and the use of steel pins as opposed to titanium pins reduced the volume of yielded bone. The latter is an interesting finding given the interest in titanium over steel for EF components, due to the lower density of titanium.

In one of the earliest studies, Behrens and Searls [18] suggested that single plane unilateral frames were found to be effective in 80% of the cases related to tibial fractures. Moreover, where additional rigidity of the frame was required, sagittal one-plane frames with double rods were preferred over either single-plane or two-plane unilateral frames. This was due to practical reasons such as better access to the wound and the less cumbersome nature in an operational sense. However, there have been many studies afterwards to investigate the effectiveness of modified versions of the unilateral EF. For complicated forms of fractures, several modified versions of the basic EF configurations given in Table 1 have been used.

Modifications to the standard unilateral EFs have been made in the forms of bilateral and monolateral EFs, based on the position of the connecting rods relative to the fractured bone. The idea of bilateral EFs has been floating amongst the medical community [21, 41]. However, its use to treat injuries has been minimal because the transfixing full pins has been found to impede the functional use of the fractured area [39]. On the other hand, the mono-lateral EF has been used extensively in treating basic fractures [42, 43, 44]. From an orthopaedic point view, its relatively simple structure, coupled with the possibility of using half pins promotes its useability [45, 46, 47]. Similarly, depending on the planes along which pins are inserted for the purpose of treatment, bi-planar [48, 49, 50] and mono-planar [51, 52, 53] variants of the unilateral EFs have previously been used. Below is a discussion on other variations of the linear, circular and hybrid EFs.

### 2.1 Linear EFs

**Fig. 2:**
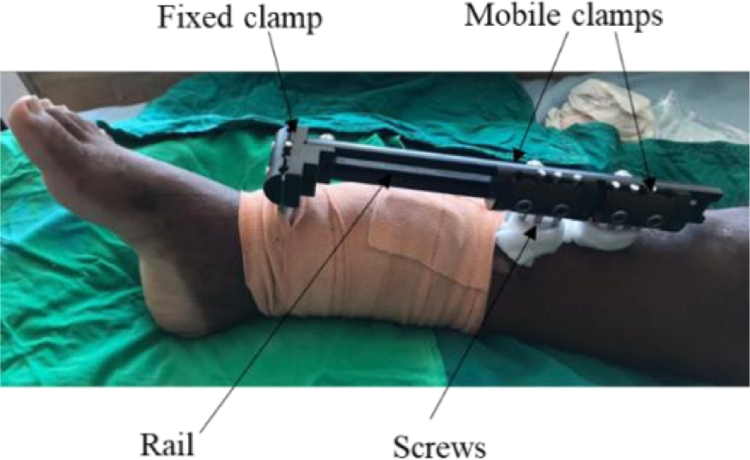
Illustration of a monorail EF.

Agrawal et al. [54] carried out a prospective study to evaluate the effectiveness of a monorail unilateral EF in treating complex femoral non-union, where the generally preferred treatment method of using ring EFs was found to be problematic. As illustrated in Fig. 2, it is an assembly of screws and clamps, generally a combination of both fixed and mobile clamps, where the latter slides on a rigid rail [55]. This type of EF is considered a Limb Reconstruction System (LRS) and is a more stable version of the unilateral EF, as well as convenient to both the patient and the surgeons. The enhanced stability can be attributed to the fact that the location of the clamps can be moved during the healing process, to ensure the screws are inserted at the most suitable locations, thus reducing undesirable movements within the EF. Moreover, in cases of non-union, LRS is considered as a salvage option when other modalities have failed, because the fracture ends can be prepared and then compressed which cannot be done in the standard EF [56]. It also allows distraction osteogenesis and thus limb lengthening and deformity correction with different types of clamps.

**Fig. 3:**
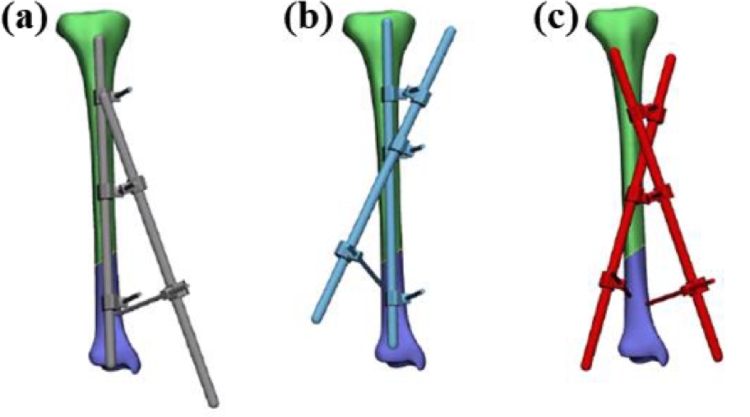
The three modified configurations of the EF with (a) no cross, (b) single-cross and (c) double-cross [3].

In treating tibial fractures, a double-cross EF configuration was found to be more effective than a no-cross or a single-cross EF configuration by Wahab et al. [3], when considering the parameters von Mises stress, displacement and relative micromotion between the fracture site and EF. The EF was assessed under a 500 N axial load, four-point bending load due to 1000 N and 15 N torsion loads. The reason for the superior performance of the double-cross EF is only attributed to its rigidity. However, a close inspection of the structural arrangement of the EF as given in Fig. 3 suggests that the angles of the pin, and the pin locations also has the potential to influence the results, which have not been discussed.

**Fig. 4:**
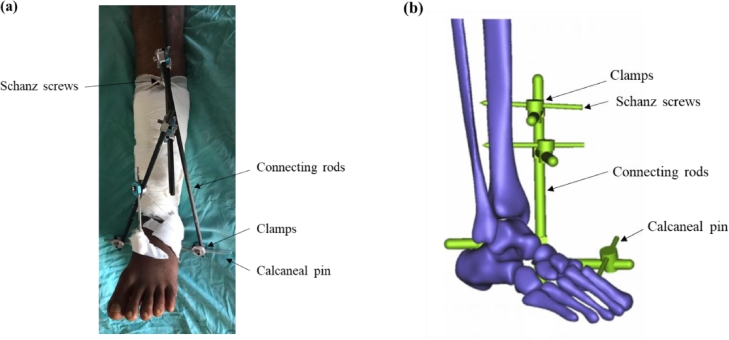
Setup of a (a) Delta and (b) Mitkovic External Fixator [59].

While stating the possibility of using the Ilizarov ring frame to treat distal tibial fractures, Ramlee et al. [57], investigated the effectiveness of a delta fixator, where the setup is as shown in Fig. 4(a). It was shown that the Delta EF had the potential to reduce the stresses at the pin-bone interface, as well as being a stable design. Patil et al. [58] also evaluated the effectiveness of a delta EF in treating distal tibial fractures and based on the outcome of clinical studies, concluded that this type of EF facilitates soft tissue healing and provides a stable fixation. Interestingly, they also go on to conclude that this type of EF can be useful when financial constraints are present, which highlights the delta EF as a viable low-cost solution. However, the reasons for this conclusion are not given and hence, requires further investigation.

In a Finite Element Analysis conducted by Ramlee et al. [59], the performance of a Mitkovic and Delta EF in treating a subtalar dislocation was compared. As shown in Fig. 4(b), the former is a modified version of the unilateral EF and provides three-dimensional freedom at each pin. The comparison was carried out for an axial load of 70 N and 250 N to represent the swing and stance phase, respectively. The delta EF yielded much superior results in terms of von Mises stress induced in the tibia, calcaneus and first metatarsal, as well EF itself, and the deflection in the EF and the bone. Although the reasons for these observations have not been mentioned, a close inspection of the two fixator setups suggest that the Delta EF possess superior distribution of stresses due to its three-dimensional arrangement of the connecting rods. Furthermore, the nature of insertion of the Calcaneal pin (also known as the Denham pin) in the Delta EF, enhances its mechanical stability, when compared to the Mitkovic EF.

Pavic et al. [60] conducted mechanical bending tests to compare the performance of a novel EF against a standard dynamic fixator, that can be used to treat tibial fractures. This novel EF was a modified version of the unilateral EF with a circulatory locking mechanism to enhance the flexibility of the fixator as well as to reduce the need for pin repositioning. The advanced mechanical testing procedure included the usage of non-contact three-dimensional optical measuring systems to measure the displacement along the three axes. Two test cases, where bone fragments in contact and with a gap of 10 mm, were conducted. Interestingly, both static and dynamic loading tests were conducted, where in the latter, a cyclic load representative of the movements of the human gait was exerted on the bone-fixator system mounted on a Servo-hydraulic testing machine. The results indicated the proposed novel EF was beneficial in terms of deflection, in cases where a gap was present between the fragments. While the reasons for this was not discussed from a biomechanical point of view, this beneficial behaviour can perhaps be attributed to the utilization of the flexibility of the EF. This is because unlike in the event where the fragments are in contact, when they are apart, the EF is able to reshape itself to the differential movement of the fragments, which can lead to less deflection.

In order to address a research gap of the absence of biomechanical studies to investigate the possibility of using EFs to treat Hallux valgus, Erdil et al. [61] designed and tested a mini-external fixator and compared its performance against a conventional lag screw fixation. The mechanical testing was carried out using a universal dynamic test system, to simulate the daily cyclic loading and the models were tested for axial compression, distraction, torsion, and bending. The proposed mini-external fixator showed superior performance in terms of the number of cycles to failure and failure load. This was attributed to the stiffness of the fixator, coupled with the possibility of controlled rotation about multiple axes, made possible by the arrangement of its pins and clamps.

Sternick et al. [20] numerically investigated the contribution of the Schanz pins towards the stiffness of a platform type dynamic EF, which consisted of a bar and two multi-planar clamps. The EF was subjected to an axial load of 200 N, which was deemed representative of the maximum load an EF will undergo during the fracture healing period. The results indicated that an EF with four Schanz pins per clamp had a higher stiffness and lower von Mises stress compared to a system with two and three pins. While the outcome of the study is very much in line with the expectation, it is interesting to investigate if there is a possibility whether the performance of the EF may be compromised if the number of Schanz pins was further increased. This is because although theoretically an increase in the number of pins per clamp will increase the overall stiffness of the construct as well as other clinical advantages such as less surgical time and less technically demanding surgery, there could be other practical implications that may arise with it. There may be a possibility of a stress riser between the pins - which can lead to higher fracture risk - if the minimum distance of bone between two adjacent pins are reduced due to the use of a higher number of pins per clamp. Also, if one pin loosens, this may affect the overall pin-clamp unit and result in destabilization of the other pins too. From a clinical point of view, possible complications such as pin-site infection and can spread rapidly to the adjacent pins, when the pins are placed close to each other. Hence, it is evident that although from an engineering point of view, a higher number of pins per clamp is preferred to withstand the loads passing through the frame, especially in the case of complex fracture and frame configurations, the practically feasible number of pins per clamp should be determined intra-operatively.

**Fig. 5:**
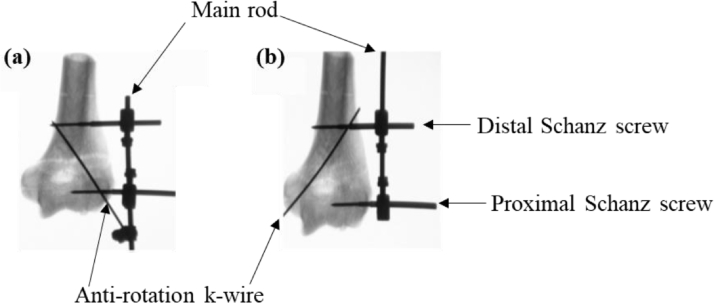
(a) The standard and (b) modified versions of the EF in treating elbow fractures [62].

Hohloch et al. [62] conducted biomechanical tests to compare the performance of an EF with a modified arrangement of the anti-rotation k-wire, in treating elbow fractures. As shown in Fig. 5(a), the standard arrangement of this type of EF is where the anti-rotation k-wire is connected to the main rod at one end and engages with both the proximal and distal Schanz screws. However, as shown in Fig. 5(b), in the proposed modification, the k-wire only engages with the distal Schanz screw. These bone-EF systems were subjected to forced-regulated cyclic loads which induced internal rotation, external rotation, extension and flexion. The proposed EF arrangement was found to give significantly favourable results in terms of reducing internal rotation, while the other factors remained largely unchanged. These observations were explained in terms of the influence of the k-wire, where in the case of internal rotations, the k-wire contributes towards the radial compression under internal rotation, but not so much under external rotation.

Elmedin et al. [63], studied the performance of an unilateral, biplanar EF named “Sarafix” in treating unstable tibial fracture. Mechanical tests were carried out, where the bone-EF system was subjected to axial, 4-point bending and torsion loads of 600 N, 500 N and 15 Nm, respectively. The deflection at the point load was measured and used to calculate the axial, bending and torsional stiffness of the fixator, as well as to validate the numerical model. The findings of the stiffness values confirmed the superior performance of the Sarafix fixator. Although the reasons for these findings have not been discussed, it can be attributed to the structure of this type of EF, where its bi-planar action can provide sufficient stiffness in preventing movements during the initial healing stages of the bone. Matsushita et al. [64] compared the effectiveness of a multi-axial correcting EF and ring EF, by conducting a clinical study. The multi-axial correcting EF was found to be effective in correcting multi planar tibial deformities.

Aziz et al. [17] carried out a numerical investigation to compare the effectiveness of three types of EFs, namely, unilateral, ilizarov and hybrid in treating femoral fractures. Based on the results of the numerical analysis the unilateral EF was found to yield the least displacement for both the EF and the bone and the lowest, yet acceptable inter-fragmentary movement (which is an indication of adequate stiffness of the EF). Moreover, the lowest von Mises stress was also in the unilateral EF, which was attributed to the higher contact area with the bones, due to the usage of pins with a higher diameter (hence lower stress), as opposed to wires with lower diameter (hence, higher stress) in the other two configurations. It was also noticed that the stress and strain at the pin-bone interface was also lowest in the unilateral configuration, which is crucial towards the stability of the EF by minimizing pin loosening as well as minimizing secondary bone-fractures, which can lead to a speedy recovery.

Pacha et al. [65] considered a rotational fixator in treating femoral shaft fractures, where control of the rotation angle, through Schanz screws or pins was a critical consideration. The outcome of the in-vitro study showed that the EF met the acceptable level of rotation required for the healing of long bone fractures, albeit with potential further improvements before used in an in vivo setting.

### 2.2 Circular and hybrid EFs

The Ilizarov EFs has been widely used to correct three-dimensional deformities, which is a significant advantage compared to the linear EFs, where other benefits include, greater stability, minimal dissection of soft tissues and a potential salvage option in scenarios where internal fixation or other EF techniques have failed [66]. This form of EF was introduced by Gavril Abramovich Ilizarov, where he discovered the phenomena of “distraction osteogenesis” when his patient turned the units to the opposite side, which led to distraction of the fracture ends. This discovery revolutionized the treatment of bone loss as this provided a technique to reform a patient's own native bone [67]. Khanfour [68] identified the potential benefits of the Ilizarov EF in treating ankle arthrodesis. Such benefits included the possibility of tailoring the frame to match the patient's requirement, enhanced circumferential rigidity thus ensuring all other movements except for axial movements are restricted and enhanced stability due to the use of pretensioned wires. The pretension wires in circular EFs have been the focus of many investigations, because the loss of pretension in the wire can lead to instability of ring fixators. Wire slippage and material yielding have been identified as potential reasons for the loss of pretension [69, 70, 71].

A retrospective analysis carried out by Lovisetti et al. [72] on the use of Ilizarov EFs in treating high energy femoral fractures, showed that it is a reliable technique in terms of its success rate and useability . As highlighted in the work, EFs are not the first choice in treating such fractures due to factors such as delayed non-union or malunion and pin infection. In the event EFs have been used, uniaxial EF devices have been the preferred configuration. Hence, this study went against the tide and proposed the Ilizarov EF. From a biomechanical point of view, the reason for this choice is the enhanced stability of the Ilizarov EF. Furthermore, the possibility of excessive joint stiffness was minimized through the correct positioning of the hinges and half pins. A previous retrospective study by O'Neill et al. [73], highlighted the lack of studies involving the use of Ilizarov EFs in treating lower limb trauma. However, both these studies clearly highlight the effectiveness of Ilizarov EFs, especially in cases where no other treatment method seems practical. Therefore, more attention in terms of biomechanical studies can be invested into Ilizarov EFs, so that its performance is better understood by all stakeholders.

Despite the many advantages of using Ilizarov EFs, its drawbacks during the application and healing stages are well documented, which include longer learning curve with a greater requirement for technical expertise, need for image intensification intraoperatively and potentially inappropriate in emergencies such as in vascular injury or compartment syndrome [74]. In addition to being bulky and cumbersome, other medical demerits such as multiple pin track infections, pin breakage, injury to opposite thigh, knee stiffness, bone osteomyelitis, distraction failure, vascular, complications and refracture have raised concerns about its suitability. Hence, there have been attempts to overcome these drawbacks through careful redesign of the Ilizarov EFs.

Grivas and Magnissallis [75] proposed twin ring Ilizarov fixator instead of the standard single ring arrangement, in treating peri‑ or intra-articular fractures of the tibia or femur, where the presence of short metaphyseal bone fragments may make the application of the latter challenging. These two systems were tested under axial and shear loading. The results of the axial loading test showed that the twin ring arrangement was less stiff than the single ring arrangement, while the pattern was reversed under the shear loading test. Both these outcomes were found to emphasise the benefits of the twin ring arrangement, because a lower stiffness under axial loading enables the required micromotion in the axial direction and the consequent compression between bone fragments, whereas a higher stiffness under shear loading is indicative of the resistance of motion in the transverse direction, which is critical to facilitate the callus formation.

Rozis et al. [76] clinically investigated the use of a combined cross screw and Ilizarov EF for ankle fusions. The cross screws induced compression at the fusion site, as well as provided enhanced stability which were found to be important contribution towards the healing process. This modified version of the Ilizarov EF was found to be of particular significance due to its weightbearing capabilities.

Qiao et al. [77] proposed a Q fixator, the name derived by its shape of an English letter “Q”, in treating lower limb long bone fracture. Due to the arrangement of pins and the connection between the two rings themselves, the fixation principle of this fixator is similar to an Ilizarov fixator. However, it is more beneficial functionally due to the possibility of incorporating computer aided design, which reduces the burden of the surgeon. The work in this paper also included an interesting work flow, where a customized Q fixator is 3D printed based on the reconstructed 3D scanned image of the fracture. Use of this advanced manufacturing technique minimized the manufacturing error to only 0.1 mm and also since the connections can be achieved without the intervention of a surgeon, the potential human error and its complications can be significantly minimized. Both these factors can contribute to expedite the fracture healing process. In this study the Q fixator was used in foam femur, cadaveric femur and cadaveric tibia fracture models, and was found to be effective in reducing the rotational deformities. Higher lateral displacement near the fracture site was observed, which was attributed to the gap between the pin and the fracture site, but can be corrected using Schanz screws.

Since the Ilizarov EF was found to be less flexible when used in cases which require complex bone reconstruction, the Hexapod EF systems have been employed as an effective tool in correcting such bone deformities [78]. Due to its structure, this type of EF is also capable of incorporating multi-axial corrections. The Taylor Spatial Frame has been the most commonly used Hexapod EF system [79, 80]. It has shown favourable correction of complicated deformities with minimal instrumental operation [64, 81]. Moreover, in the case of distal femoral open comminuted fractures, the Hybrid EFs have produced good outcomes, when compared to the Ilizarov EFs [82]. Caja et al. [83] compared the performance between linear, circular and hybrid EFs and found that axial, bending and torsional stiffness was highest for the linear fixator. However, the hybrid fixator showed stiffness values closer to that of the linear fixators, thus, making them a viable candidate in treating complicated forms of fractures.

The preceding detailed discussion highlights the many novel developments with respect to the structural configurations of EFs. Where applicable, the behaviour of these different EFs have been explained from an engineering point of view. Further optimization of EFs must also consider the contribution of different materials, which will be discussed in the next Section.

## 3 Commonly used materials in EF structural systems

The choice of materials is a key consideration when designing and fabricating EFs. A balance needs to be maintained between biocompatibility that ensures non-interference with the healing process and structural integrity leading to overall stability of the EF. To this end, the choice of materials has shifted from pure metals and its alloys to composites such as carbon fibre reinforced plastic and metal polymer composites [16, 84, 85]. Several studies have been carried out by designing the entire EF using a single metallic alloy. Such studies have used steel grades such as AISI316L and V_4_A and titanium grades such as Ti_6_Al_4_V. Several studies have highlighted the superiority of stainless steel over titanium, mainly due to the higher yield strength and elastic modulus of the former, thus making it a better choice from a biomechanical point of view [86, 87, 88]. However, electrochemical and biological inertness of titanium makes it an attractive candidate from a medical point of view, especially in the case of pins [89, 90]. Polymer composites have also been preferred for EF components due to its mechanical properties such as high strength to weight ratio, higher fatigue strength compared to metals, higher ductility compared to ceramic composites, conformability to anatomical shape and elasticity [91, 92].

In recent years, nanomaterials have gained significant interest in the field of orthopaedics [93]. The contribution of nanomaterials towards EFs has been twofold, namely, preservation of biocompatibility and enhancing of structural integrity. The former has been the focus of most studies, especially in minimizing pin tract infections [94, 95]. Lyons et al. [96] highlighted the potential of nanostructured piezoelectric polymers for EF components such as screws and pins due to its advantageous mechanical properties such as high strength and impact resistance.

A review of the different materials used for the main parts of the EF, namely main bar, connecting rods, clamps and pins, will be beneficial for future studies. The present Section reviews some of the commonly used materials for EFs and their usefulness.

### 3.1 Connecting rods

Stainless steel has been the most frequently used material for the connecting rod [97], [98], [99], [100]. Radke et al. [101] stated that in order to increase the mechanical stiffness of connecting rods without a significant increase in the weight, materials such as titanium, aluminium and carbon fibre are preferred over the widely used stainless steel rod, which is in fact a view shared by others [60]. As discussed below, several studies have compared the performance of EFs, where the connecting rods have been designed using the aforementioned materials.

Frydrýšek et al. [102] proposed the use of carbon fibre as opposed to titanium connecting rods in treating pelvis fractures. Added antibacterial protection, partial visibility in case of x-rays, lower stress and deflection and lower weight of the carbon fibre rods were the reasons behind this proposition. Tomanec et al. [103] carried out a finite element analysis to evaluate the performance of a ring fixator in treating tibia fractures, where the connecting rods and supporting rings were designed using carbon fibre composites and the rest of the components were designed using titanium and stainless steel. The EF with carbon fibre composites showed a 63% reduction in weight compared to a fully metal EF. The results of the finite element study showed that the maximum allowable stress in the carbon fibre composites was 1.72 times more than the maximum generated stress. Pervan et al. [97] compared the performance of unilateral, biplanar Sarafix EFs, where the connecting rods were fabricated out of Carbon Epoxy and Stainless steel (grade X30Cr13). The EFs with Carbon Epoxy rods showed 44% decrease in the stress. In another study, Pervan et al. [104] used E glass/epoxy resin, Kevlar 49/epoxy resin and Carbon M55J/epoxy resin as the connecting rod materials of an EF similar to the previous study and compared them against an EF with a stainless steel rod. Of the three composite EFs, the Carbon fibre composite showed the best mechanical properties, while the glass composite displayed the worst. These four configurations were numerically analysed for their axial, bending and torsional stiffness and the EF with the carbon fibre composite connecting rod was found to possess the highest stiffness in all three cases. Ong et al. [105] used a carbon fibre connecting rod with a density of 1500 kg/m^3^ and stainless steel pins in a Hoffmann II MRI external fixation system and this system was found to be a stable arrangement. Hence, these studies provide evidence for the feasibility of carbon fibre composites as the material of choice for the connecting rod.

### 3.2 Clamps

While earlier designs of EFs have used steel and titanium clamps, recent versions have considered composite materials of lower weight. Basat et al. [40] carried out a quantitative statistical analysis for metallic alloys and polymers to determine the most suitable material for the clamps of an unilateral EF. The metallic alloys which were considered were AISI316L, Ti_6_Al_4_V, Aluminium 7175 and Magnesium AZ80A-T5, whereas high-density polyethylene, Polyethylene terephthalate, Polyether ether ketone and Polycarbonate were the polymers considered. The density, tensile strength, ultimate strength and price were the factors considered for metallic alloys while, melting temperature, glass transition temperature, density, tensile strength, ultimate strength and price were considered for polymers. The analysis revealed that the titanium alloy Ti_6_Al_4_V and Polyethylene terephthalate were the most suited metal and polymer, respectively. Landaeta et al. [106] developed a novel linear fixator, where the clamps were 3D printed using a composite material containing nylon with chopped carbon fibre randomly distributed in the filament. During the biomechanical testing, this clamp arrangement was found to provide sufficient rigidity with minimum shrinkage. Therefore, these recent developments encourage further research on alternative lightweight materials for the clamps.

### 3.3 Pins

The pin material also plays a critical role in the performance of the EF and the healing process of a fractured bone. Presently, the commonly used pin materials are stainless steel and the titanium alloy Ti-6Al-4 V. The main reason for the choice of these materials is their higher strength. However, in general, both steel and titanium possess high elastic moduli, former in the range of 200 GPa and the latter in the order of 100 GPa [107]. These values are significantly higher than the elastic modulus of bones which is generally in the range of 20 GPa [107]. Hence, this apparent disparity between the elastic moduli of the pin material and the bone can lead to stress concentrations at the pin-joint interface due to lower recoverable deformation. Hence, there is a need to identify suitable materials for pins, where a material with a lower elastic modulus, without compromising on the strength, is required. Zheng et al. [107] compared the performance of pins in a unilateral EF, where the pins were made of two titanium alloys, namely Ti2448 and Ti_6_Al_4_V. The former has a comparable or a slightly higher strength, while possessing a significantly lower elastic modulus (by 70%). However, the elastic modulus of Ti2448 (33 GPa) is closer to the elastic modulus of that of a cortical bone (20 GPa). When subjected to compression, bending and torsional tests, EFs with pins made out of both these titanium alloys indicated a smooth variation between stress and strain, which was indicative of a stable EF system. However, the EF system with Ti2448 pins indicated higher recoverable deformation, which highlighted its superior performance. Moreover, pin loosening, which significantly hampers the healing process due to excessive micromotion at the pin-joint interface was also found to be less in the Ti2448 pins. Hence, this study concluded that the Ti2448 alloy promises to be a viable substitution to the existing pin materials. As for Schanz screws, stainless steel and titanium [53, 89, 108] have been used in previous EF designs.

### 3.4 Rings in circular EFs

The largest element in a circular EF is the ring component. Previous studies suggest that steel, titanium aluminium and carbon composites have been the preferred materials [109, 110]. Kalova et al. [111] proposed the use of carbon fibre reinforced epoxy resin based polymer composite for the rings in an Ilizarov EF. The high specific modulus and strength of carbon fibre reinforced plastic at both room and elevated temperatures, coupled with the permeability offered by the resin, which is beneficial for X-rays, made this composite a viable option. Lobst [112] identified braided carbon fibre as a new material that is being used in rings, due to its higher strength to weight ratio and possessing higher radiolucent properties compared to the traditional materials.

The preceding discussion indicates the shift of focus from the traditional metallic alloys to light weight composite materials when designing EFs. It can also be observed that the advancements in the manufacturing techniques have complemented this transition. However, it is evident that there is considerable space to further improve optimized selection of materials, which will be further discussed in Section 5.

## 4 Experimental and numerical biomechanical tests to evaluate the EFs

Biomechanical investigations of an EF system are a mandatory requirement, prior to its use in clinical studies. While experimental investigations are ideal and reliable, due to complexities such as finding the appropriate test setups to replicate actual loading scenarios and fractured bone structures, expensive nature of tests etc. there has been a shift towards investigations using finite element modelling. With the advancement of computational resources, conducting numerical simulations to replicate the bone-fixator interaction has been made feasible. Modelling of overall geometry, materials properties and contact definition between the bone and fixator components are some of the main considerations, in order to increase the accuracy of the numerical simulations. This Section reviews the current level of knowledge with regards to both experimental and numerical investigations of EFs.

### 4.1 Experimental investigations of EFs

**Fig. 6:**
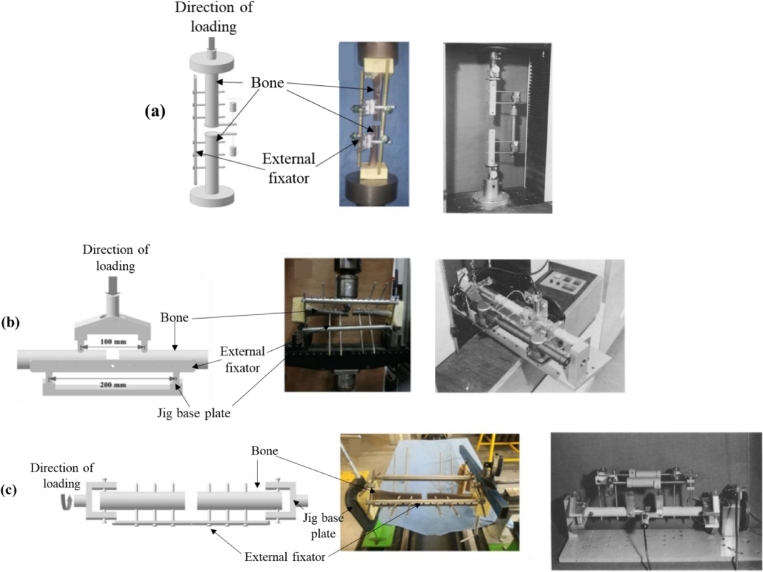
Experimental setups used for (a) axial, (b) bending and (c) torsional tests [49, 114, 115].

From a practical point of view, the elements in an EF can be subjected to axial, bending and torsional loads during its use. For example, when considering the anchorage pins, flexural and torsional loads are decisive, whereas for clamps, the flexural effects are critical. On the other hand, the rings in the circular fixator must be evaluated for compressive axial loads, due to the pretention in the wires connected to them. When considering an EF-bone arrangement, axial, bending and torsion loads can be relevant and previously used experimental setups for these tests are provided in Fig. 6. Therefore, a biomechanical evaluation of the EF must take into account these three loading scenarios and this can be found in ASTM F1541–17, “Standard Specification and Test Methods for External Skeletal Fixation Devices” [113]. This standard itself has undergone many changes and its present format provides testing methods for connecting elements such as clamps, in-plane compression of ring elements, joints, anchorage pins, subassemblies and external fixator-bone constructs. However, a method to test the entire assembled fixator is yet to be approved.

INSTRON uniaxial testing machines have been used to conduct the axial compression tests. Generally, the bone-EF setup has been loaded up to the range of 700 N, which is the corresponding weight of a human weighing 70 kg in a one-legged stance [49, 114, 116]. The loading rate for these tests are generally in the order of 10^−4^ m/s, which falls under the static range [114]. The bending tests have been carried out as either three point or four bending tests on several testing machines, where jigs have been used to provide the required offset for the force, in order to generate the moment about the axis of interest [114, 117, 118]. The offset value used in previous studies is generally in the range of 50 mm, where the load has been applied at a rate of 0.5 mm/s, with the bending moment considered in the range of 20 Nm. As for torsional tests, many customized jigs have been employed in previous studies [114, 116, 117]. The maximum angular displacement has been in the range of 10°/s, with increments of 0.5°/s and the maximum torque value has been in the range of 5–10 Nm, with increments of 0.2 Nm. These tests have previously been carried out to determine the strength as well as the stiffness of the EF. While the strength can be readily determined using the experimental output, the axial, bending or torsional stiffness has been calculated as the slope of the linear portion of the load vs displacement curve obtained through the corresponding test [115, 119]. In cases where a non- linear behaviour was observed in the load vs displacement curve, four axial stiffness values were calculated for the four portions corresponding to 25% intervals of the maximum load, while two values were calculated for both the bending and torsional stiffnesses for the two portions corresponding to 50% of the maximum load [83]. Hence, it is evident that such experimental biomechanical investigations have provided a basis to compare the performance between different types of EFs. [83, 116, 120, 121]. As identified in Sections 2 and 3, since various forms of EFs can be constructed using different structural forms and materials, the experimental measures identified in the preceding discussion provides the most reliable basis of determining the behaviour of a given EF system.

### 4.2 Numerical investigations of EFs

According to Sternick et al. [36], the first finite element analysis of an EF was conducted in 1977. Since then, numerous numerical studies have been carried out to study the behaviour of EFs.

Defining accurate material properties of the human bones can be considered as one of the major factors in numerical modelling, as it directly contributes towards modelling the behaviour of the bones when subjected to a load. Table 2 summarizes the mechanical properties of the human body parts that have been used in numerical simulations, along with the material definitions employed in these numerical simulation software. As evident by the findings, most studies have not considered the inhomogeneous nature of the bones, but instead have used simplified definitions such linear isotropic. Donaldson et al. [37] identified the oversimplification of the behaviour of bones by using isotropic material properties to simulate them and hence, uses orthotropic properties in their study. This enabled a more accurate method of capturing bone-yielding, where a strain-based approach was preferred over a stress-based approach due to relatively minor variations of the yield strain in anisotropic bones.

**Table 2:** Summary of key definitions previously used in finite element modelling of EFs.

Body parts	Defined parameters	Material Definition	Contact definitions	Ref
Cortical and cancellous bones	Young's modulus, Poisson's ratio	Homogeneous and linear-isotropic	Fully bonded connection between Cortical and cancellous bones and a 0.3 friction coefficient between EF-bone interface	[3]
Cortical bone, marrow and granulation tissue	Young's modulus, Poisson's ratio	Homogeneous material properties	Tie constraint between bone and pins to replicate clamps	[4]
Cortical and trabecular bone	Young's modulus, Poisson's ratio	Homogeneous material properties	Rigid fixation in the contact area with the knee, and free in the foot connection, where the loads are applied in a region of 20% equivalent to the bottom surface of the tibia	[5]
Cortical and cancellous bones for Osteoporosis and osteoarthritis conditions	Young's modulus, Poisson's ratio	Linear isotropic behaviour	Friction coefficient of 0.4 was used to represent the contact condition between the pin of the external fixator and bone	[57]
Cortical and cancellous bones	Young's modulus, Poisson's ratio	isotropic, homogenous, and linearly elastic	The interface between cortical and cancellous bone layer was assumed as fully bonded while partially bonded between the fixator and bone	[17]
Cortical and cancellous bones and ligaments	Young's modulus, Poisson's ratio, Stiffness	isotropic, homogenous and linear	The contact body between the external fixator and the bone was set with a friction coefficient of 0.4 based on a previous study. Contact between bones was set with a friction coefficient of 0.3.	[59]

Table 2 also provides details of the different contact definitions employed in the numerical models. Two main definitions are the contact between the Cortical and cancellous bones and bone and EF pin. In most cases the former is defined as a fully bonded connection, while the latter is defined using a friction coefficient. However, in reality both these connections must be defined using friction coefficients and specifically, static and dynamic friction coefficients, if both static and quasi-static loads are considered.

Another important consideration in Finite Element Modelling is the quantification of load. In simulating the behaviour of the bone-EF arrangement under axial, bending and torsion loads, Wahab et al. [3] considered loads of 1500 N, 500 each at the proximal tibia and distal tibia and 15 N at the proximal tibia, respectively. Kolasangiani et al. [4] considered 25% of the body weight as the axial load to be applied. Considering the average mass of a person as 75 kg, Ebrahimi et al. [122] considered an axial load of 1500 N and 3000 N for a subclinical and clinical level assessment. In order to simulate human walking conditions, Ramlee et al. [123] considered two axial loads, corresponding to what is referred to as the “swing phase” and “stance phase” of a gait cycle. 10% of the body weight was found to be a reasonable approximation for the former, while 50% of the body weight was considered for the latter. Hence, it is clear that there is no clear agreement on the magnitude of the loads under which the EF-bone arrangement must be assessed. Moreover, while most studies have only focused on static analysis of the bone-EF system, Roseiro et al. [5] considered the dynamic effect of loading by taking into account the modes of vibration. However, the dynamic analysis was limited to comparing the stiffness of the system, as opposed to considering the actual dynamic response of the system.

Karunratanakul et al. [124], carried out a comprehensive numerical validation of the performance of a unilateral EF in treating a rabbit tibia fracture. Prior to the mechanical testing of the bone-EF system, the non-destructive axial compression testing was conducted on the bones, to determine their axial stiffness, based on the force and deflection readings from the experiments. Similarly, the bending stiffness of the bone was determined by applying a known load at a known distance, to a screw which was drilled through the bones. The bone-EF system was then axially loaded up to a compressive force of 100 N and the interfragmentary displacement was measured. These same arrangements were numerically modelled using MSC Marc 2010 (MSC Software Corporation, USA), where the bone geometry was developed using radiography. The boundary conditions of the system were defined to be representative of the actual test conditions. The behaviour of the bone was assumed to be linear-isotropic, and the numerical model of the bone was first validated using the axial and bending stiffnesses obtained experimentally. As for the final numerical model with the bones and EF, the main aim of it was to identify the appropriate contact setting at the screw-bone interface and the correct estimation of the stiffness of the bone-EF system. The study by Kolasangiani [4] using ABAQUS, where the numerical model was validated by comparing the axial stiffness of the bone-fixator in the model and the actual unit employed in the in-vitro study. Elmedin et al. [63] validated the numerical model by comparing the load vs displacement values of the EF for axial, bending and torsional tests and then used the validated numerical model to analytically calculate the displacement of the proximal and distal segments of the fracture gap along the three principal axes. These results were used to calculate the axial, bending and torsional stiffness of the fracture. It is interesting that this numerical study has chosen to use the material properties of wood to define the orthotropic behaviour of the bones. While the comparability of properties between wood and specific human bones are debatable, this approach of defining material properties in ways other than isotropic, can be considered an approach worth further investigation. Li et al. [125] carried out a Finite Element Analysis using ANSYS, to complement the analytical model developed to calculate the axial, bending and torsional stiffness of a unilateral EF. This study identifies the applicable loads and boundary conditions when carrying out the analysis to compute the aforementioned stiffness values.

Similar comprehensive work flow in developing a validated numerical model can also be found in [126], [127], [128]. Amaro et al. [126] developed a detailed numerical model by replicating key information such as the geometry, connections, constraints, friction factors and pin stiffness of the EF system used in the experimental setup. The numerical simulations were carried out using the finite element software ADINA software and the validation of the numerical model was performed by comparing the load vs deflection values from the experiment. Mesic et al. [127] carried out the numerical simulations using the finite element software CATIA, where the validation of the numerical model was based on the magnitude of the principal stresses at the mid-point of the connecting rod, at varying compressive loads. Zhao et al. [128] used the finite element software ANSYS to simulate the behaviour of the bone-EF construct during four stages of fracture healing (or growth states), when subjected to axial, bending and torsional loads. The variation of the material properties as well as the boundary condition of the bones during the four stages of healing was incorporated to the numerical model by separate definitions for each stage. The validation of the numerical model was carried out by comparing the strain of the bone-EF construct for each case of loading, during each stage of healing. In all three of these studies, preliminary numerical investigations have been carried out to identify the most suitable element formation and mesh size, which significantly contribute to the accuracy of the results.

The above discussion highlights the importance of complementing the finite element models with experimental proof.

## 5 Analysis of the review- Conclusions and future directions

The preceding Sections discussed the existing level of knowledge in terms of the available structural systems, usage of materials and biomechanical performance of external fixators. Based on the review of literature, the following research gaps were identified and are highlighted for the benefit of future studies.

Most studies have only studied the performance of EFs under static loading. However, in reality, the load exerted during standard human activity involving change of posture is more quasi-static or dynamic in nature. Whenever, dynamic loading has been considered in previous studies, it has been idealized as a cyclic sinusoidal load. Therefore, this only takes into account intentional dynamic loads. However, there can be much severe unintentional dynamic loads experienced by a person wearing an EF, where the load with a high magnitude may act for a small period of time. Therefore, the biomechanical tests must also incorporate provisions to induce these loads onto the test setup. The applicable magnitude of such loading as well as the rate of loading can be decided with the inputs from orthopaedic surgeons. Moreover, there is considerable disagreement over the magnitude of loads even when evaluating the EFs under static loads, and the input from orthopaedic surgeons will be beneficial on this regard too. For example, from an orthopaedic point of view, while the circular EFs can be expected to be full weight bearing in certain cases, the linear fixators are generally non-weight bearing. Therefore, all these factors must be taken into account when deciding on the applicable load to evaluate the performance of EFs.

A careful inspection of the published literature suggests that the treatment of material behaviour, whether it is in the EF or in the bones has been at a primitive level. For example, when metals or metallic alloys have been used in EFs, only the behaviour within the elastic range of the materials has been considered. However, it is common knowledge that beyond the yield strength materials transition into an inelastic zone, where parameters such as ‘stiffness’ and ‘strength’ cannot be limited to their definitions within the elastic range. Hence, disregarding the behaviour of materials in the inelastic zone can lead to unrealistic results. This is especially applicable in the recent EF designs, where relatively low strength non-metallic composites such as Polymers are used to fabricate certain parts.

As evident in Section 4.2, homogeneous material properties have been assigned to the bones. Although this has been identified as an over-simplification, numerical studies have failed to propose a method to consider the inhomogeneous nature of bones. The anisotropic behaviour of bones, which is the dissimilarity between key mechanical properties along the three principal directions, demands special attention when defining material properties. The advanced non-linear finite element software commonly used today includes material definitions which can take into account the anisotropic behaviour of the bones. This needs to be complemented with material testing to obtain relevant parameters along the principal axes. Also, the properties of bones vary with factors such as gender and age and readily available data on such factors can contribute towards well informed decisions by orthopaedic surgeons.

Although there is a considerable number of studies related to the behaviour of pins, certain aspects have yet to be conclusively determined. For example, determination of the number of pins which will contribute to an optimum level of stiffness in the EF system and alternate material with high strength and low elastic modulus can be listed as two such aspects.

It was also observed that there is a lack of comprehensive studies which consider both experimental and numerical aspects. While numerical modelling has shown to be a reliable technique, it must be complemented with experimental evidence. Therefore, more studies which considers both these aspects must be encouraged.

From a fabrication point of view, it can be seen that only a limited number of studies have made use of the advancements in the manufacturing techniques such as 3D printing and cold spraying. Moreover, further studies can be carried out to investigate the structural integrity of EFs fabricated using nanomaterials. These techniques can pave the way for customized EFs, which can minimize most complications. Moreover, conducting a cost-benefit analysis of fabricating different types of EFs using potential materials will be useful, especially in manufacturing low cost EFs. Furthermore, such studies will be able to identify the most cost-effective material and manufacturing technique for each component of the EF, which when incorporated into conceptual models such as the one developed by Sellahewa et al. [129],can lead to better optimization and customization of EFs in the future.

## Funding

This research was funded by the National Institute for Health Research (NIHR) (Grant Number: 16/137/45- NIHR Global Health Research Group on POsT Conflict Trauma; PrOTeCT) using UK aid from the UK Government to support global health research. The views expressed in this publication are those of the author(s) and not necessarily those of the NIHR or the UK Department of Health and Social Care.

## Ethical approval

Not required.

## Declaration of Competing Interest

None declared.
